# Editorial: Reviews in serious games and mobile health interventions: form design, implementation, user engagement, and behavior change

**DOI:** 10.3389/fpubh.2026.1903161

**Published:** 2026-07-07

**Authors:** Costina-Ruxandra Poetar

**Affiliations:** 1 Department of Clinical Psychology and Psychotherapy, Babeş-Bolyai University, Cluj-Napoca, Romania; 2 The International Institute for the Advanced Studies of Psychotherapy and Applied Mental Health, Babeş-Bolyai University, Cluj-Napoca, Romania

**Keywords:** implementation, interventions, mobile health, serious games, virtual reality

## Introduction

Serious games are effective in improving health outcomes. They show benefits for particular mental health problems (1), chronic conditions (2) or memory issues for older adults (3). Mobile health interventions, including mobile applications, wearables, and digital interventions, have also demonstrated effectiveness in alleviating health problems (4), improve management of diabetes and obesity (5), address mental health (6), support medication adherence (7), and increase physical activity (8).

Serious games and mobile health interventions can overcome gaps in treatment access and deliver evidence-based interventions in a standardized manner. While their effectiveness has been demonstrated across a wide range of health outcomes and populations, identifying factors related to implementation, design, user engagement, and behavior change can further enhance their effectiveness.

This Research Topic seeks to synthesize existing evidence on serious games and mobile health interventions, with particular emphasis on implementation characteristics, sustained behavior change, and integration within current healthcare systems.

## Overview of contributions

The present issue includes 10 articles with different designs: four systematic reviews, four cross-sectional studies, one randomized controlled trial (RCT), and one conceptual framework for conducting interdisciplinary reviews. Ciemer, Klotzbier et al. propose a cross-disciplinary literature search framework that could increase sensitivity and robustness compared to discipline-specific searches. The topics from this issue are diverse, covering interventions (remotely delivered, virtual rehabilitation, combat sports, virtual reality [VR] cycling, digital interactive and game-based fall interventions) or investigations into factors related to telemedicine, AI-generated content, and internet hospital pharmacy adoption.

### Factors influencing adoption intentions

Liu J. et al. investigated the motivations behind adults' adoption of AI-generated content during health information searches. Performance expectancy was a key factor across all configurations tested, underscoring its importance to the adoption of AI-generated health content.

Liu C. et al. examined the relationship between online patient trust and telemedicine behavior among 582 Chinese adults. Results showed that online patient trust emerged as a key predictor of telemedicine behavioral intention. The findings suggest cooperation between the government, healthcare providers, and patients can reduce risks, increase credibility, and strengthen guarantees.

### Impact of digital interventions on health-related outcomes

Zhao et al. present results from an RCT comparing a VR intervention to a control intervention (traditional cycling). Results indicated that participants in VR cycling with music group reported better outcomes (weight, BMI, waist and hip circumferences, vital capacity). The sample consisted of 78 college students from China.

Li et al. reviewed combat sports (e.g., boxing, martial arts, judo) in VR for rehabilitation and physical activity. From the 18 included studies, VR Boxing emerged as the most frequently studied intervention, with positive therapeutic outcomes in patients with neurological conditions. These include improvements in motor function, balance, trunk control, and quality of life.

Ciemer, Kröper et al. conducted a systematic review analyzing existing digital fall interventions for healthy community-dwelling older adults. The review included 59 studies, analyzed using qualitative thematic synthesis across four areas: objectives, design and development, types of interventions, and evaluation methods. Results show screen-based VR is most used, with mixed reality also common. The authors call for more cross-disciplinary collaboration to address the identified gaps.

Tian et al. carried out a systematic review and meta-analysis to evaluate the effectiveness of digital interventions for adult asthma. Results of the meta-analysis, based on 10 studies, indicated moderate effects on quality of life at post-test and small-to-moderate effect sizes at 12-month follow-up compared to usual care.

Arora et al. provide an overview of Natural User Interfaces, multisensory interfaces, and user experience design strategies in applications and devices specifically developed for individuals who are partially or completely visually impaired. The authors extracted information regarding their usability, universal usability, user feedback, and identified challenges.

### Implementation evaluation and healthcare integration

Klein et al. employed a mixed-methods design grounded in contribution analysis to examine a smartphone-based citizen science program aimed at preventing gender-based violence in rural Tanzania. Their study enabled a comprehensive assessment of the intervention's impact and implementation.

Gao et al. investigated the characteristics and safety of whole-course-based Internet hospital outpatient pharmacy services. Participants were 351,884 patients who received medication in 2020 and 2023. Results indicated significant reductions in costs and days, low error rates, and high patient satisfaction.

The development of well-defined regulations and standardized quality and safety frameworks is necessary to support the adoption of digital tools and to guide policymakers in their implementation.

### Barriers in implementation and future research

The implementation of digital health interventions is hindered by a combination of design, accessibility, and engagement-related challenges that cut across technologies and user groups. A key issue is the mismatch between intervention formats and user needs. High attrition rates and variability in engagement across technologies (e.g., VR vs motion-based platforms) are important challenges (Tian et al.; Li et al.). Personal factors, including sex differences in response to VR interventions, further emphasize the need for personalized interventions (Zhao et al.).

Usability and accessibility barriers persist due to limited device functionality, poor compatibility with available services, and insufficient customization (Arora et al.). Financial constraints, low scalability, and the gap between prototype development and market availability further restrict adoption. Across studies, users consistently highlight affordability, personalization, adaptability, and ease of use as critical facilitators, yet current solutions frequently fall short in these areas (Tian et al.; Zhao et al.).

Research should also place greater emphasis on user perspectives rather than assuming that facilitating conditions will drive adoption (Klein et al.). Future research must identify successful implementation factors through RCTs, with longer follow-ups and the use of mixed-methods, clinician-rated outcomes, rather than relying solely on self-report, to overcome the high risk of bias.

## Conclusion

This Research Topic contributes to understanding the factors that drive engagement in health interventions across diverse populations. The findings demonstrate how digital interventions can extend the reach of health interventions, provided that usability, credibility, and contextual barriers are appropriately addressed. The contributions also emphasize inclusivity, demonstrating that personalized design, whether for older adults, individuals with disabilities, or underserved communities, is essential for assuring equitable access and sustained engagement.
